# The Impact of Team Learning Climate on Innovation Performance – Mediating role of knowledge integration capability

**DOI:** 10.3389/fpsyg.2022.1104073

**Published:** 2023-01-17

**Authors:** Ming-Shun Li, Jiaqi Li, Jia-Mei Li, Zi-Wei Liu, Xin-Tao Deng

**Affiliations:** School of Traffic and Transportation Engineering, Changsha University of Science and Technology, Changsha, China

**Keywords:** team learning climate, knowledge integration capability, innovation performance, empirical analysis, mediating effect

## Abstract

To address the fierce competition for corporate innovation in the digital economy, this study introduces knowledge integration capability as a mediating variable in light of social information processing theory, and explores the mechanism of team learning climate on innovation performance. Data were collected from a sample of 184 team members for statistical analysis, and Statistical methods such as descriptive statistical analysis, correlation analysis, and regression analysis were used to verify the study hypotheses through SPSS and Amos software, and the results showed that: (1) Team learning climate has a significant positive effect on knowledge integration capability. (2) Team learning climate has a significant positive effect on innovation performance. (3) Knowledge integration capability has a significant positive effect on innovation performance. (4) Knowledge integration capability partially mediates the role between team learning climate and innovation performance. The results proved the perspective of knowledge integration capability for the mechanism of team learning climate on innovation performance from the perspective of knowledge integration capability, and provided theoretical references for creating a learning climate in companies to promote members’ knowledge learning and enhance innovation performance.

## 1. Introduction

With the advent of the digital economy, a new generation of information technology such as cloud computing and artificial intelligence has emerged, driving enterprise production toward intelligence and automation. The development of the digital economy is constantly shortening the validity of knowledge, compressing the cycle of new product development, and promoting fierce competition among companies. Enterprise innovation driven by the digital economy needs to support team members to share knowledge and effectively utilize and integrate the corresponding knowledge to enhance their innovation capabilities.

Today, some scholars’ studies have explored the mechanisms of team supportive contextual factors on members’ innovation performance, and it is generally agreed that supportive contexts can promote continuous learning for employees, thus enhancing innovation performance (Tripathi and Kalia, 2022). Team learning climate is defined as a shared perception of team members that the organization promotes, supports, and rewards their learning behaviors (Peng and Chen, 2022). It has been suggested that the learning climate is a precursor to producing valuable outcomes (Cangialosi et al., 2020) and an important potential mechanism for innovative behavior (Pigola and Da Costa, 2022). There are few corresponding studies on the mechanisms of team learning climate on innovation performance. Therefore, this study will systematically examine the impact of team learning climate on innovation performance.

The ability to utilize existing knowledge and information to produce different combinations and reconfigurations is the source of innovation (Liu and Chan, 2017), which is also known as knowledge integration capability. By continuously creating a climate for learning, the team encourages the exchange of knowledge and ideas among its members and enhances their knowledge integration, which, in turn, promotes the generation of new knowledge and innovation (Lau and Ngo, 2004). It has been pointed out that the stronger the knowledge integration capability, the stronger the ability of the company to develop new products, respond to new situations, and enhance creativity (Wang et al., 2018). Knowledge integration facilitates teams to quickly identify new opportunities, assimilate internal and external knowledge, and then reorganize and innovate knowledge to enrich the existing knowledge base, contributing to innovative products (Gong et al., 2022). Therefore, this study will introduce knowledge integration capability to explore the mediating mechanism between team learning climate and innovation performance.

In summary, this study establishes a conceptual framework based on previous findings and theoretical gaps found in the literature, and investigate the relationship among team learning climate, knowledge integration capability and innovation performance, and the role of knowledge integration capability in the above mechanisms from the following aspects: Firstly, we analyze the impact of team learning climate on innovation performance. Then, knowledge integration capability is incorporated into the research framework to analyze the impact of team learning climate on knowledge integration capability and the impact of knowledge integration capability on innovation performance, and to explore the mediating role of knowledge integration capability. Finally, data from company employees were collected by distributing an electronic version of the questionnaire, and linear regression analysis and other methods were used to verify the hypotheses and draw conclusions.

The value of this study is to explain the direct relationship between team learning climate and innovation performance and to analyze the mediating role of knowledge integration capability. In brief, the findings complement the mechanism of team learning climate on innovation performance and suggest that team learning climate plays a positive role in innovation performance; expand the research perspective of knowledge integration capability, which plays a partially mediating role; and provide a new direction for the improvement of innovation performance in Chinese SMEs.

## 2. Literature background

### 2.1. Team learning climate

Research on climate emerged in the late 1960s and became common in fields such as organizational psychology and organizational behavior (Schneider et al., 2011). Schneider believes that climate is the common perception of policies and procedures in an organization that can be easily observed and measured (Schneider, 1990). Since then, research on organizational climate has extended to many types, among which the impact of learning climate on the development of adaptive capacity and the ability of individuals and teams to cope is crucial (Westerberg and Hauer, 2009). Nikolova defines learning climate as the common perception of employees about organizational policies that support, promote and reward learning behaviors (Nikolova et al., 2016). And when employees perceive that the organization supports them in their efforts to learn on the job, they are more likely to actively interact and learn to accomplish their tasks (Hirak et al., 2012). Previous research supports this idea. Armstrong argues that a prerequisite for organizations to generate significant outcomes is the construction of a learning environment that enhances employees’ intent to learn and encourages their active participation in learning activities (Armstrong-Stassen and Schlosser, 2011). Kyndt found that organizations showing prior support for learning behaviors can enhance employees’ intentions to learn, improve existing knowledge and skills, and opportunities to develop new knowledge and skills (Kyndt et al., 2013).

Despite the theoretical emphasis by scholars on the importance of learning climate, empirical studies on how team learning climate promotes innovation performance are still lacking. Previous studies on organizational climate on innovation performance have focused on supportive climate and rules climate (García-Buades et al., 2015), innovation climate (Waheed et al., 2019) and knowledge hiding climate (Haar et al., 2022). The mechanism of influence of team learning climate is not very clear.

### 2.2. Knowledge integration capability

knowledge as a static resource that cannot be used directly. Only within the team through understanding, absorption and memory can knowledge rise from perceptual awareness to rational thinking, thus forming innovative thinking (Alavi and Leidner, 2001). Knowledge integration is not a simple superimposition of knowledge, but a dynamic coupling of knowledge elements through the dynamic flow of acquired knowledge within the team (Mehrabani and Shajari, 2012). Mehrabani proposes a model of knowledge integration based on knowledge absorption, where he argues that knowledge integration can filter foreign information and resources and reorganize their use after understanding them.

With the popularity of capability theory, some scholars have tried to elevate the concept of knowledge integration to the level of organizational capability. Kogut believes that knowledge integration capability is the ability to use existing knowledge to reorganize and innovate (Kogut and Zander, 1992). Ramesh summarizes knowledge integration capability as the ability of organizations to combine the knowledge possessed by individual members and reorganize it into the knowledge needed for a specific goal (Ramesh and Tiwana, 2001). Based on the characteristics of the digital economy, Liu argues that in the era of information explosion, knowledge integration capability is the ability to filter, process, and reorganize various fragments of information and resources inside and outside the team and perform knowledge innovation (Liu and Du, 2018).

In general, although scholars have interpreted knowledge integration capability in a large number of ways based on different perspectives, their understanding of the meaning of knowledge integration capability is basically the same. It is all about acquiring various types of knowledge and then reorganizing and even innovating them through the members’ understanding and absorption to ensure the core competitiveness of the organization.

### 2.3. Innovation performance

As one of the important ways to solve problems and maintain competitive advantage, scholars have been paying great attention to the research about innovation. Muller believes that innovation can lead to new products, new services, and other results, which in turn can improve business performance, market returns (Muller and Peres, 2019). Ustalar defines innovation performance as the synthesis of the output performance of a company using knowledge technologies in innovation activities in daily production operations (Ustalar and Şanlisoy, 2020). On the other hand, some scholars consider the set of activities that produce something new as innovation, and innovation performance as all the outcomes that result from this process. For example, Li believes that knowledge accumulated during the innovation process can enhance innovation performance, and such accumulated knowledge is itself an invisible innovation outcome (Li et al., 2018). To sum up, this study summarizes innovation performance as the results of new ideas, new models, new products, and new technologies generated in the innovation process.

## 3. Hypothesis development

### 3.1. Team learning climate and knowledge integration capability

The digital economy distinguishes itself by its focus on the flow of digital resources and the emphasis on value co-creation. That is to say, it lays, the emphasis on knowledge flow, knowledge exchange and knowledge sharing. In a high team learning climate, the team is keen to create an environment conducive to knowledge exchange and knowledge sharing for its members, provides learning opportunities, and encourages team members to collaborate and communicate with each other and actively solve problems on a continuous basis (Eldor and Harpaz, 2016). Based on social information processing theory, the behavior and activities of individuals are influenced by the external environment in addition to their individual needs (Pfeffer, 1978). With a strong team learning climate, team members’ inner desire for knowledge grows stronger and they realize the importance of knowledge for work and innovation, thus enhancing their knowledge integration capability.

Knowledge is held by individuals and if knowledge mobility and knowledge innovation are desired, existing competencies should be reorganized to learn new knowledge and skills (Kogut and Zander, 1992). Harvey argued that once a team learning climate is formed, members agree that continuous learning and self-development are team goals, and they motivate members to actively engage in learning behaviors (Harvey et al., 2019). Meanwhile, the conflict between individual and team interests is weakened by the material and spiritual incentives of the team, which enhances the willingness to share knowledge among members and continuously motivates them to acquire and innovate knowledge (Gara Bach Ouerdian et al., 2017). Therefore, team learning climate, as a shared perception, has a positive impact on both team members’ willingness to share knowledge and their capability to integrated knowledge.

Based on the discussion, the following hypothesis is proposed:

*H1*: Team learning climate is positively correlated with knowledge integration capability.

### 3.2. Team learning climate and innovation performance

Through the learning and utilization of information and knowledge, the actions of team members, such as applying new thinking, proposing new models or developing new products, can enhance the core competitiveness of the team and bring intangible and tangible economic benefits to the team, the company and society (Hwangbo et al., 2022). Some scholars have found that team learning climate offers members opportunities for knowledge exchange, feedback, and helps them make deeper connections between their work and team goals (Cangialosi et al., 2020). In such a climate, members perceive their roles and tasks to be fluid among themselves and other team members, and easily changed through mutual learning. As a result, members can easily handle their work based on the knowledge and skills they have learned from their team members. In addition, team learning climate provides greater opportunities and challenges for members, fostering a sense of accomplishment and emotional attachment to the team such as a sense of identity and responsibility (Eldor and Harpaz, 2016). These positive emotions motivate members to remain rational in the face of problems and believe that they can accomplish their goals (Gable and Dreisbach, 2021). At this point, team members demonstrate excellent extended thinking, which enhances innovation performance.

Teams in high learning climate are conducive to creative participation of members. In team learning climate, the team provides emotional support, technical support, and creative support for members’ innovative learning activities, and members feel a sense of organizational support, which enhances their intrinsic motivation and leads to members’ active engagement in innovative behavior, goal accomplishment, and their overcoming of difficulties and challenges without fear of using creativity (Cerasoli and Ford, 2014), thus improving the innovation performance. According to self-determination theory, high team learning climate in which members feel organizational support enhances members’ intrinsic drive and sense of responsibility, thus prompting members to aspire to be in continuous positional challenges in improving themselves. Meanwhile, resource conservation theory states that team learning climate as a resource, members with more of this resource will be more actively engaged in their work to better preserve and acquire resources, such as conducting innovative activities to gain innovation performance to form a positive spiral structure of resource accumulation (Inkpen, 1996).

Based on the discussion, the following hypothesis is proposed:

*H2*: Team learning climate is positively correlated with innovation performance.

### 3.3. Knowledge integration capability and innovation performance

The development of the digital economy has made information exchange easier, reduced the cost of searching for information, and accelerated knowledge sharing, which consequently increases the efficiency of transforming knowledge into innovative products (Lyytinen et al., 2015). According to the knowledge-based theory of the firm, the maintenance of a firm’s core competitive advantage depends on the efficiency of its teams in transforming knowledge, information and technology (Crescenzi and Gagliardi, 2018). Knowledge integration capability can help teams promote the rapid flow, sharing, application, and innovation of knowledge among members, and improve the understanding and utilization efficiency of external knowledge as well as internal members’ knowledge (Martini et al., 2017). When members’ knowledge integration capability is enhanced, they can easily integrate external fragmented knowledge and their own knowledge to reorganize and innovate into a new knowledge system, which lays the foundation for members’ innovative behavior. Moreover, when facing sudden environmental changes and conflicts, knowledge integration capability can help enhance technical strength, broaden knowledge stock, and motivate members and teams to conduct product innovation and market planning more efficiently (Zobel et al., 2017).

Domestic and foreign scholars have made numerous studies on knowledge integration capability, and generally agree that knowledge integration capability and innovation performance are positively correlated. Ritala argues that integrating expertise among team members in order to adapt to a specific context allows teams to plan products more efficiently to facilitate product innovation (Ritala et al., 2017). Moreover, Wang argued that teams have good performance in innovation projects such as technological innovation if they are able to acquire new knowledge and integrate old knowledge that already exists within the team, i.e., the stronger the knowledge integration capability, the better the team innovation performance (Zhao, 2022).

Based on the discussion, the following hypothesis is proposed:

*H3*: knowledge integration capability is positively correlated with innovation performance.

### 3.4. The mediating role of knowledge integration capability

In the face of open innovation in the digital economy, the high-speed flow of knowledge workers promotes the interaction of information, while the important way for team members to improve the efficiency of their own innovation is precisely to enhance the ability to utilize information and learn from knowledge (Akcigit and Kerr, 2018). The access of team members to knowledge and the strength of their knowledge integration skills are also influenced by the learning climate and the external knowledge environment (Boh and Wong, 2013). Members in high team learning climate can more easily gather and decode information that can be converted and innovated into knowledge, expertise and decisions (Cauwelier et al., 2019). Companies rely on these members to help teams think beyond existing inertia, better assess the value of new information in a specific field, selectively choose new knowledge and skills based on the needs of the innovation, reduce uncertainty about the innovation, and give practical meaning and application to the innovation product (Men et al., 2018).

Based on the definition and role of knowledge integration capability and scholars’ researches, this study combined hypothesis H1: team learning climate is positively related to knowledge integration capability, and hypothesis H3: knowledge integration capability is positively related to innovation performance, and inferred that team learning climate may enhance innovation performance by improving team members’ knowledge integration capability.

Based on the discussion, the following hypothesis is proposed:

*H4*: Knowledge integration capability play a mediating role in team learning climate and innovation performance.

### 3.5. The hypothesis model

**Figure 1:**
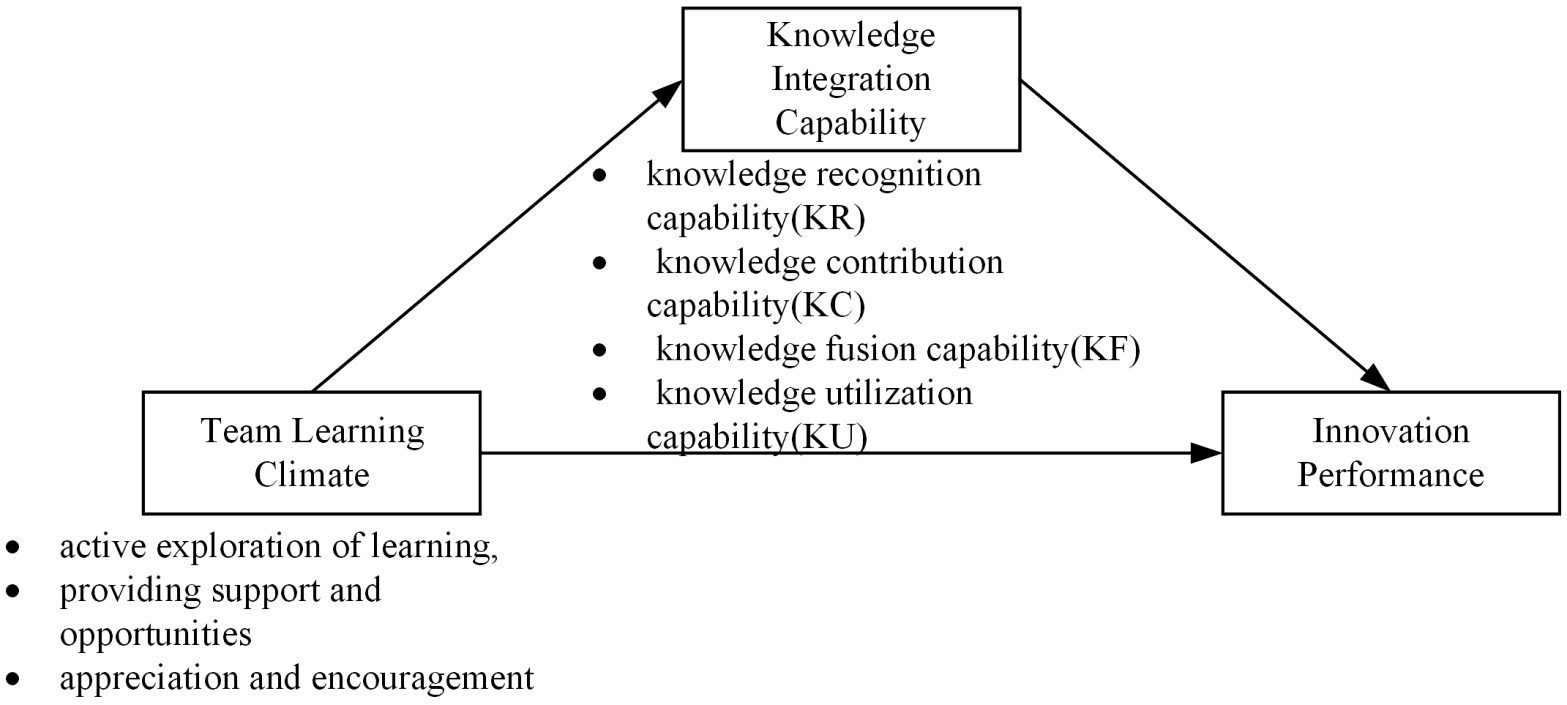
The hypothesis model.

In summary, the theoretical model of this study is shown in Figure 1.

## 4. Methodology and data analysis

### 4.1. Participants and procedures

The purpose of this study is to examine the relationship among team learning climate, knowledge integration capability, and innovation performance. In order to obtain a larger and more representative sample, employees working in R&D technology, market research and market planning were selected. The procedures were as follow: To begin with, we found a contact person for each position in each company to clarify the purpose and content of the questionnaire to reduce their resistance to the study. An online questionnaire was then sent to each participant detailing the study and the anonymization system, and the participants were asked to carefully review the questions and reply.

Therefore, in this study, questionnaires were sent to 208 employees in September 2022, who answered questions on the control variable, the independent variable (team learning climate), the dependent variable (innovation performance), and the mediator (knowledge integration capability). The returned questionnaires were analyzed to eliminate incomplete or inconsistent questionnaires, and 184 valid questionnaires were retrieved, with an effective rate of 88.46%. Demographic data revealed that: the sample was composed mainly of women (66.3%), compared to (33.7%) of man. The predominant age profile was 18 to 30 years old (76.6%), while the proportion of the participants over 50 years old was only 1.92%. The sample size of undergraduate and graduate and above accounted for 67.9 and 25.0% respectively, which indicates that the respondents generally have a high level of education. In the working years, the sample size of those who worked for less than 3 years accounted for 79.3%.

### 4.2. Measures

The measures used in this study was to design a questionnaire based on existing established scales, using the principle of a 5-point Likert scale, with five levels from 1 to 5 representing “strongly disagree” to “strongly agree” respectively.

#### 4.2.1. Team learning climate

A six-item scale designed by Spara (2007) was used to classify team learning climate (TLC) into active exploration of learning, provision of support and opportunities, appreciation and encouragement to reflect employees’ perceptions of team learning climate. Respectively items, such as “Members of this team spend a lot of time learning new things,” “The top management of this team really supports team members’ efforts to develop ourselves” and “Members of this team get rewarded for acquiring new skills.” The mean coefficient alpha was 0.819.

#### 4.2.2. Knowledge integration capability

The scale designed by Liu and Du (2018) was used, which is subcategorized into Knowledge Recognition Capability (KR), Knowledge Contribution Capability (KC), Knowledge Fusion Capability (KF) and Knowledge Utilization Capability (KU). The scale contained a total of 12 items, such as “Our team members are well aware of the knowledge and skills we possess,” “Our team members have access to the knowledge needed from relevant materials within the team,” “Our team members have the ability to reassemble internal knowledge for new product development,” and “Our team members are able to apply new knowledge to solve new problems.” The mean coefficient alpha was 0.873.

#### 4.2.3. Innovation performance

Using the innovation performance (IP) scale complied by Janssen (2000). The scale has 9 items such as “Members often translate new ideas into useful practice” and “Members often come up with creative solutions to problems.” The mean coefficient alpha was 0.879.

#### 4.2.4. Control variables

Considering the previous studies, five control variables (gender, age, education background, working years and working position) were selected.

## 5. Results of data analysis

### 5.1. Data analysis

For the data analysis and the validation of the hypotheses, regression analysis, which requires a low number of data samples, was used. To determine the minimum sample size for the linear regression model of this study, calculations were carried out using the PASS software designed by NSCC with reference to the tables and theory provided by Cohen (1988) and Gatsonis and Sampson (1989). The results indicated that the minimum sample was 50. Therefore, a total of 184 valid samples were obtained for this study, which could be analyzed by linear regression. In linear regression, a linear relationship between the independent and dependent variables is required; the errors obey a normal distribution with a mean of zero; and there is no collinearity between the variables. The variables in this study met the above requirements.

The basic process of the testing is divided into the following steps: firstly, the variables such as gender, age, education, and years of work are controlled for. Secondly, the reliability and validity of the factors in the measurement scales were examined to assess the quality of the model. Finally, a hypothesis testing is performed, in which the significance test of mediating effects is done by Bootstrapping.

In this study, the sample data were analyzed using SPSS and Amos software, which are among the most common software used to perform measurement model quality tests and regression analyses.

### 5.2. Measurement model

As mentioned above, this study first analyzed the reliability of the items of the measurement scale. And this was done by calculating the Cronbach’s α and Corrected Item-Total Correlation (CITC). The Cronbach’s α was originally proposed by Nunnally and Bernstein (1994) and has a minimum allowable value of 0.7. Loiacono et al. (2002) proposed to remove items whose elimination would improve Cronbach’s α by checking the CITC value, which has a minimum value of 0.4. In the pretest questionnaire, the CITC value for question item KR4 in the knowledge integration capability was −0.091 and the correlation was only 0.07, so the deletion of KR4 was considered, and the Cronbach’s α for the knowledge integration competency improved from 0.858 to 0.873 after the deletion of KR4. As shown in Table 1, the Cronbach’s α for the variables analyzed were all greater than 0.7, indicating that all variables were reliable.

**Table 1:** Results of reliability test.

Variables	Items	CITC	KMO	Sig.	Cronbach’s α after deletion of item	Standardized α	Treatment
Team learning climate			0.839	*p* < 0.001		0.819	
	TLC1	0.683			0.769		Reservation
	TLC2	0.592			0.785		Reservation
	TLC3	0.606			0.782		Reservation
	TLC4	0.526			0.8		Reservation
	TLC5	0.567			0.791		Reservation
	TLC6	0.525			0.8		Reservation
Knowledge integration capacity			0.878	*P* < 0.001		0.858	
Knowledge recognition capacity	KR1	0.473			0.829		Reservation
	KR2	0.458			0.83		Reservation
	KR3	0.555			0.823		Reservation
	KR4	−0.091			0.872		Deletion
Knowledge contribution capability	KC1	0.614			0.821		Reservation
	KC2	0.589			0.821		Reservation
	KC3	0.624			0.817		Reservation
Knowledge fusion capability	KF1	0.499			0.827		Reservation
	KF2	0.58			0.821		Reservation
	KF3	0.525			0.825		Reservation
Knowledge utilization capacity	KU1	0.59			0.82		Reservation
	KU2	0.526			0.825		Reservation
	KU3	0.59			0.82		Reservation
Innovation performance			0.894	*P* < 0.001		0.879	
	IP1	0.619			0.867		Reservation
	IP2	0.647			0.864		Reservation
	IP3	0.597			0.869		Reservation
	IP4	0.67			0.861		Reservation
	IP5	0.575			0.871		Reservation
	IP6	0.643			0.864		Reservation
	IP7	0.69			0.859		Reservation
	IP8	0.695			0.859		Reservation

Furthermore, the variables were examined by KMO test and Bartlett’s spherical test for suitability for factor analysis, that is, to test whether each variable is independent of the other. KMO test is used to check the correlation and bias correlation among variables. When KMO value is above 0.8, it means that the sample size is sufficient. And when Sig. <0.05 (*p* < 0.05), it means that the data are spherically distributed and the variables are independent of each other to some degree, and factor analysis can be performed(Vogt and Johnson, 2011).

**Figure 2:**
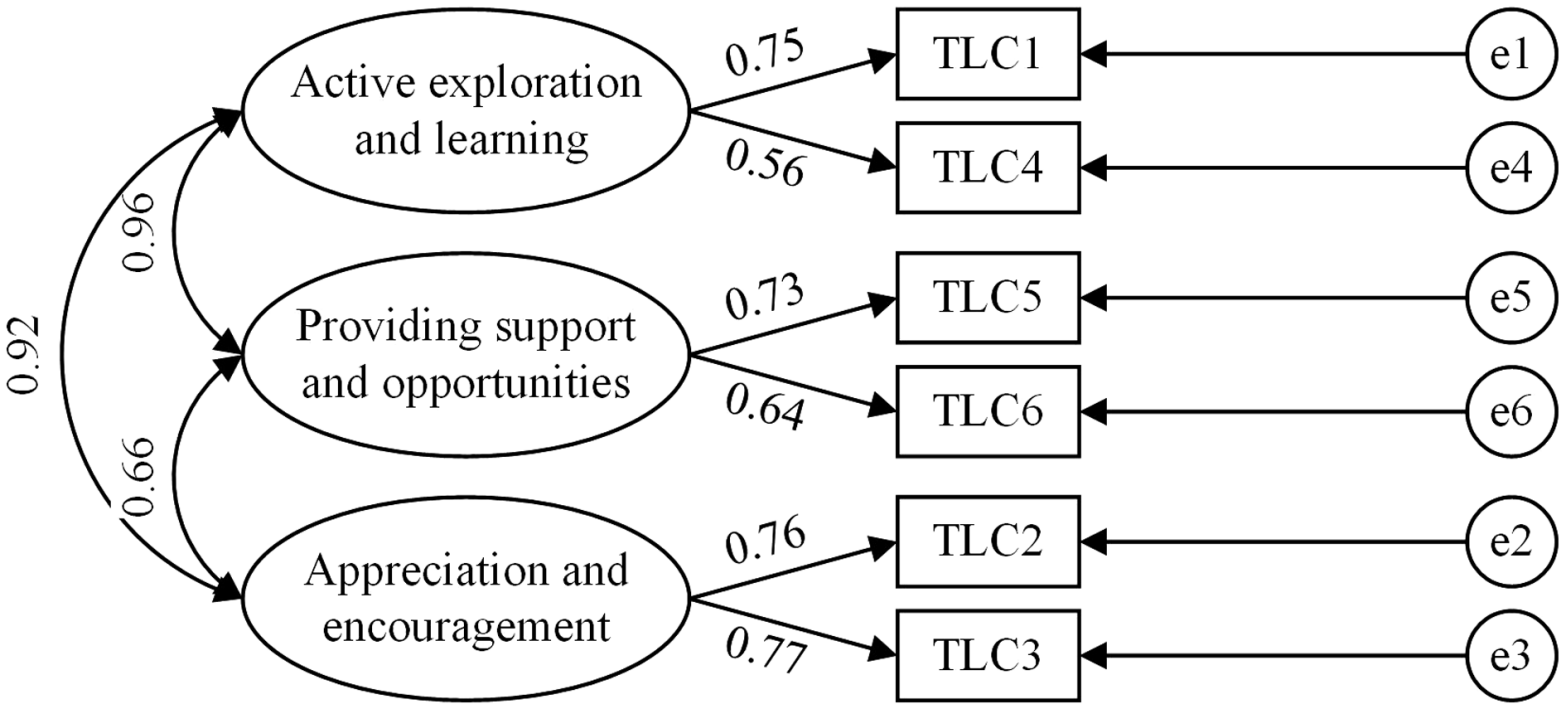
Confirmatory factor analysis model of Team Learning Climate.

**Figure 3:**
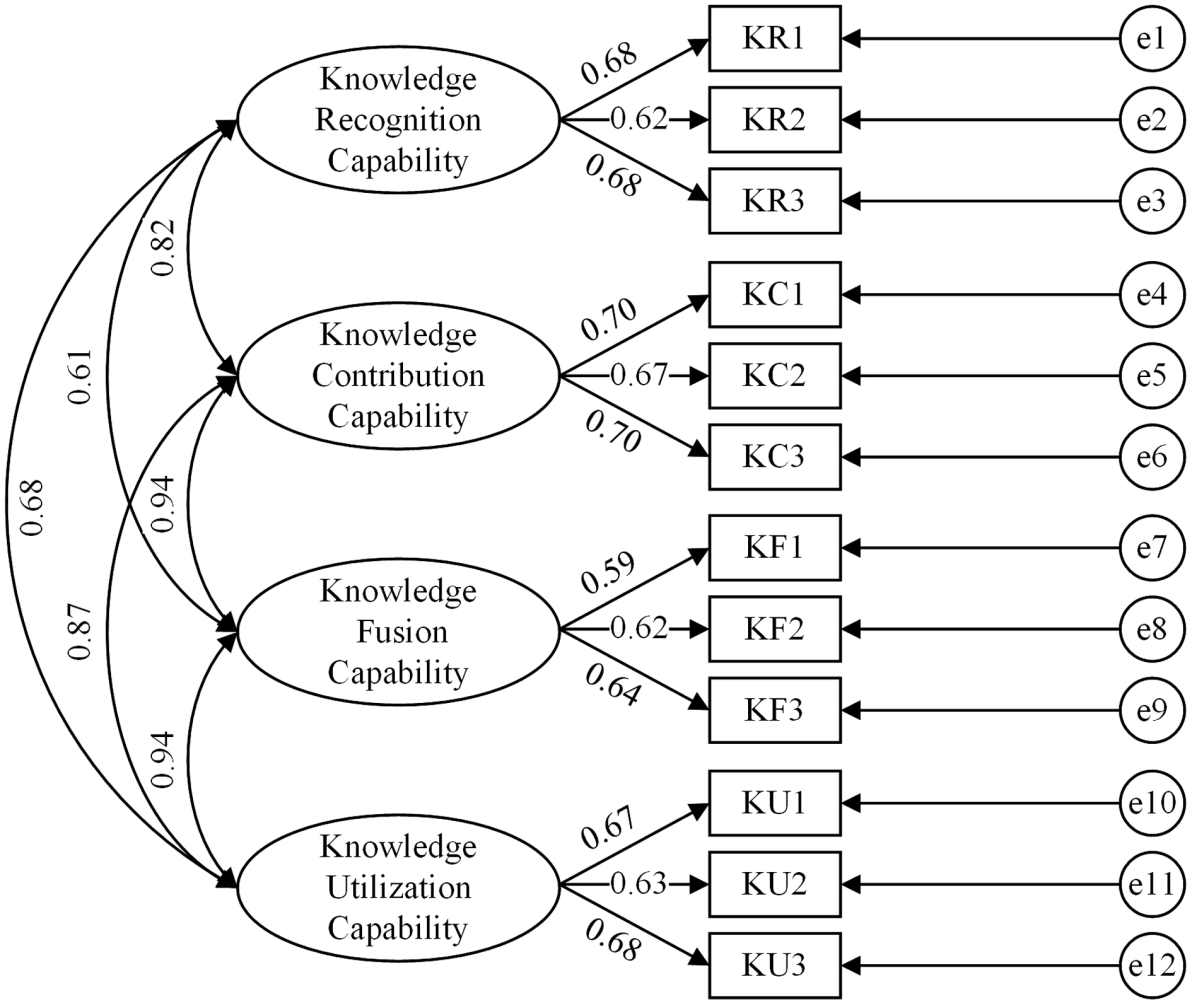
Confirmatory factor analysis model of Knowledge Integration Capability.

**Figure 4:**
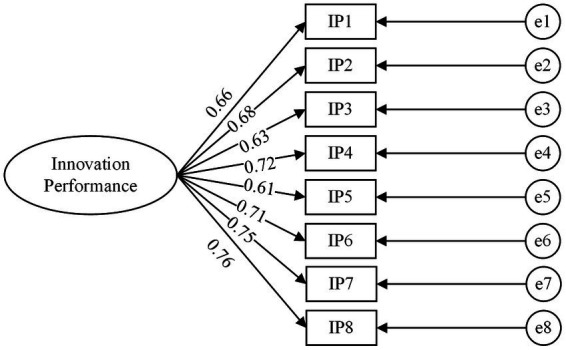
Confirmatory factor analysis model of Innovation Performance.

After the reliability analysis was completed, this study adopted confirmatory factor analysis (CFA) to assess the validity of all variables. All variables were loaded onto their respective latent variables (team learning climate, knowledge integration capability, and innovation performance), as shown in Figures 2–4. Wen (Zhong-Lin et al., 2004) considered that the model fitted well when the variables satisfied a criteria of χ2df > 3, *p* < 0.001, GFI > 0.8, CFI > 0.8, NFI > 0.8, AGFI > 0.8, IFI > 0.8, RMSEA < 0.1, RMR < 0.5. The results of CFA are shown in Table 2.

**Table 2:** Fitting index of confirmatory factor analysis.

Variables	$\chi^{2}$	χ2df	GFI	CFI	NFI	AGFI	IFI	RMSEA	RMR
Team learning climate	3.672	0.612	0.993	0.993	0.989	0.977	1.000	0.003	0.018
Knowledge recognition capability	103.576	2.518	0.915	0.923	0.868	0.862	0.924	0.080	0.081
Innovation performance	51.024	2.511	0.935	0.948	0.918	0.883	0.949	0.092	0.041

### 5.3. Descriptive statistics and correlation analysis

Before the hypothesis testing, this study conducted the necessary descriptive statistics and correlation analysis on the basic characteristics of the data. The results of descriptive statistics and Pearson correlation analysis for each variable are shown in Table 3. Team learning climate is positively associated with innovation performance (*r* = 0.662, *p* < 0.01) and with innovation performance (*r* = 0.703, *p* < 0.01). Knowledge integration capability is positively associated with innovation performance (*r* = 0.680, *p* < 0.01). This preliminarily verifies the relevant hypothesis of this study. Meanwhile, the correlation coefficients were all greater than 0.5 and less than the allowed value of 0.75 for multicollinearity (Tsui et al., 1995). As a result, this study concluded that there is no serious collinearity among the variables and that regression analysis could be performed on the data.

**Table 3:** Results of correlation.

Variables	Correlation	Innovation performance	Team learning climate	Knowledge recognition capability
Team learning climate	Pearson Correlation	1.000		
Knowledge recognition capability	Pearson Correlation	0.622**	1.000	
Innovation performance	Pearson Correlation	0.703**	0.680**	1.000

**p < 0.01.

### 5.4. Hypothesis testing

The hypotheses were tested with the help of regression model constructed by empirical methods. The measured variables were processed and then included in a linear regression model to analyze the specific relationships among the different variables, from which the significance was examined to determine whether the hypotheses were valid. In this section, the effect of team learning climate on knowledge integration capability, innovation performance, is first examined. Secondly, to test the effect of knowledge integration capability on innovation performance. Finally, the mediating role of knowledge integration capability between team learning climate and innovation performance is verified.

The results of the analysis are shown in Table 4, where team learning climate positively influenced knowledge integrate capability (*β* = 1.132, R2 = 0.462, *F* = 156.522, *p* < 0.001), team learning climate positively influenced innovation performance (*β* = 1.132, R2 = 0.462, F = 156.522, *p* < 0.001), knowledge integrate capability positively influenced innovation performance (*β* = 1.132, R2 = 0.462, F = 156.522, *p* < 0.001). Therefore, H1, H2, H3 were verified.

**Table 4:** Results of Regression analysis.

Variables	Knowledge recognition capability		Innovation performance		Innovation performance	
	β	T	β	T	β	T
Team learning climate	1.132	12.511***			0.896	11.905***
Knowledge recognition capability			0.571	13.342***		
*R*	0.462		0.494		0.438	
Adj. *R*^2^	0.459		0.492		0.435	
*F*	156.522***		178.003***		141.722***	

**p < 0.01, ***p < 0.001.

The mediating role was verified by using the hierarchical regression method proposed by Baron and Kenny (1986). We first introduce the mediating variable (knowledge integration capability) based on the assumption that H2 is valid, and test the regression coefficient magnitude of the independent and mediating variables and whether they are significant. As can be seen in Table 5, the positive effect of the independent variable (team learning climate) on the dependent variable (innovation performance) remains significant (*β* = 0.462, *p* < 0.001). However, the regression coefficient β decreases from 0.896 in Table 4 to 0.462 in Table 5, and the effect is significantly weaker. And the mediating variable (knowledge integration capability) positively affects innovation performance (*β* = 0.383, *p* < 0.001). Therefore, knowledge integration capability plays a partial role in team learning climate and innovation performance. H4 was verified.

**Table 5:** Regression analysis of mediating effects.

Variable types	Variables	Innovation performance	
		β	T
Independent variable	Team learning climate	0.462	5.060^***^
Mediating variables	Knowledge recognition capability	0.383	6.982^***^
Regression model index	*R*	0.557	
	Adj. *R*^2^	0.552	
	*F*	113.831^***^	

***p < 0.001.

The significance of the mediating role of knowledge integration capability was further verified by Bootstrap test. Setting up repeated sampling from the original sample 5,000 times, the results in Table 6 show that: The 95% confidence interval was [0.2598, 0.6441], and the interval did not contain 0. Thus, the mediating role of knowledge integration capability is significant, and H4 is further tested.

**Table 6:** Results of Bootstrap program.

Direct effect	SE	T	95% Confidence interval	Indirect effect	SE	95% confidence interval
0.4620^***^	0.0752	11.9047	[0.7471,1.0439]	0.4335***	0.0983	[0.2598,0.6441]

***p < 0.001.

## 6. Discussion

The digital economy has reduced the cost of information acquisition, search and replication (Goldfarb and Tucker, 2019), and accelerated the flow of knowledge, leading to the need for extremely strong knowledge integration mechanisms for enterprise innovation. In this case, how to actively build team climate and encourage team members to integrate knowledge is the key to improve innovation performance. In view of this, this study constructed a theoretical model of team learning climate – knowledge integration capability – innovation performance to analyze the influence of team learning climate on innovation performance and the mediating role of knowledge integration capability.

### 6.1. Theoretical implications

It has been confirmed that building supportive contexts can enhance innovation performance (Wu et al., 2019), but the specific mechanism of team learning climate as a supportive context on innovation performance has been less studied. Therefore, according to the characteristics of team learning climate that support members in knowledge sharing (Schein, 2004), we investigate the relationship between team learning climate and innovation performance from the perspective of knowledge integration capability.

The results of the study indicate that team learning climate positively influences innovation performance. This is similar to the findings of Montani et al. (2023) that when organizations express a high level of support for innovation, firm innovation performance is ultimately enhanced. When the team signals to its members that the team encourages and appreciates their learning behavior and provides them with the corresponding emotional, technical and resource support, the team members can perceive the environment, actively integrate external information, broaden their knowledge and generate new ideas, skills and methods, which in turn strengthens their ability to cope with the new and increasingly competitive situation and enhances their creativity. This study examines the impact on innovation performance through the perspective of team learning climate, which is conducive to expanding the study of factors influencing innovation performance.

Knowledge integration capability partially mediates the relationship between team learning climate and innovation performance. This suggests that team learning climate can have both a direct positive impact on innovation performance and indirectly enhance innovation performance through knowledge integration capability. Previous research has discussed the impact of individual perceived team learning climate on creativity based on resource conservation theory (Eldor and Harpaz, 2016), which provides some basis for an in-depth study in this research. This study verified the mediating role of knowledge integration capability in team learning climate and innovation performance based on social information processing theory. The findings enrich and deepen the study of mediating factors in the influence mechanism of team learning climate on innovation performance.

### 6.2. Practical implications

With the arrival of the digital economy era, China implements the innovation-driven development strategy, and drives enterprises to invest in innovation and enhance their core competitiveness. Open innovation thinking can help companies to quickly conduct innovative R&D, get rid of overcapacity and adapt to complex environments. On the basis of the results of the empirical study, the main contributions, innovation and practical implications introduced in this study are as follows:

First of all, in accordance with the digital economy’s emphasis on knowledge flow, knowledge exchange and knowledge sharing, managers should promote the climate of team knowledge sharing and form a resource base of team knowledge stock by carrying out external conditions such as seminars, academic sharing and professional knowledge lectures. In addition, the learning behavior among members is appreciated and motivated, for example, by specifying innovation-related indicators in the assessment system, so as to strengthen members’ perception of the team learning climate as an environmental factor.

Second, knowledge integration capability is divided into four dimensions: knowledge recognition, knowledge contribution, knowledge fusion and knowledge utilization, and enhancing knowledge integration capability requires comprehensive enhancement of these four dimensions. Knowledge recognition helps to target the most valuable information and knowledge to the team in the information explosion. Knowledge contribution refers to the process and behavior of knowledge holders to provide and create knowledge, and knowledge contribution among members helps to improve the whole knowledge base and facilitate others to learn knowledge. Knowledge fusion can help transform the absorption of external information and knowledge for your own use. Knowledge utilization is most important in practice, and the practical application of knowledge to new ideas, new technologies and new products is what completes the act of innovation. Therefore, managers should focus on the development of knowledge integration capability to enhance innovation performance.

Third, during their working processing, team members should focus on communication and interaction with other members and actively build and maintain relationships in order to establish good relationships within the team members. Guarantee the effectiveness of communication between managers and team members in order to build a harmonious and united team climate. This facilitates team members to devote themselves to creative work and achieve a qualitative change and leap in team creativity.

### 6.3. Limitation and future research

There are certain limitations in this study. Firstly, the cross-sectional data we took could not clarify the causal relationship among the variables. Therefore, future studies can use longitudinal studies to determine the causal relationships among variables. Secondly, although this study strives for sample diversity in sending questionnaires to collect data on knowledge workers in different regions of China, it may still produce sampling errors that make the samples more similar in some characteristics. This limits the universality of the findings, and the results may not be applicable to all types of team members. In the future, thus, the sample size can be further expanded to examine the effects of different types of corporate environments, to achieve diversified data collection, and to improve the universality and application value of research findings. Finally, the knowledge integration capability in this study only plays a partial mediating role, which indicates that there are other mediating factors in the influence mechanism of team learning climate on innovation performance. Other mediating factors can be searched for and studied in more depth in the future.

## Data availability statement

The raw data supporting the conclusions of this article will be made available by the authors, without undue reservation.

## Author contributions

M-SL and JL proposed the research topic and designed the questionnaire. JL authored and revised the manuscript. M-SL and J-ML reviews the manuscript. J-ML, Z-WL, and X-TD conducted the questionnaire and collected and organized the data. All authors contributed to the article and approved the submitted version.

## Funding

This work was supported by the National Natural Science Foundation of China (No. 52204202).

## Conflict of interest

The authors declare that the research was conducted in the absence of any commercial or financial relationships that could be construed as a potential conflict of interest.

## Publisher’s note

All claims expressed in this article are solely those of the authors and do not necessarily represent those of their affiliated organizations, or those of the publisher, the editors and the reviewers. Any product that may be evaluated in this article, or claim that may be made by its manufacturer, is not guaranteed or endorsed by the publisher.

## References

[ref1] Akcigit U. Kerr W. R. (2018). Growth through heterogeneous innovations. J. Polit. Econ. 126, 1374–1443. doi: 10.1086/697901

[ref2] Alavi M. Leidner D. (2001). Review: knowledge management and knowledge management systems: conceptual foundations and research issues. MIS Q. 25:107. doi: 10.2307/3250961

[ref3] Armstrong-Stassen M. Schlosser F. (2011). Perceived organizational membership and the retention of older workers. J. Organ. Behav. 32, 319–344. doi: 10.1002/job.647

[ref4] Baron R. M. Kenny D. A. (1986). The moderator-mediator variable distinction in social psychological research: conceptual, strategic, and statistical considerations. J. Pers. Soc. Psychol. 51, 1173–1182. doi: 10.1037//0022-3514.51.6.1173, PMID: 3806354

[ref5] Boh W. F. Wong S. S. (2013). Organizational climate and perceived manager effectiveness: influencing perceived usefulness of knowledge sharing mechanisms. J. Assoc. Inf. Syst. 14, 122–152. doi: 10.17705/1jais.00326

[ref6] Cangialosi N. Odoardi C. Battistelli A. (2020). Learning climate and innovative work behavior, the mediating role of the learning potential of the workplace. Vocat. Learn. 13, 263–280. doi: 10.1007/s12186-019-09235-y

[ref7] Cauwelier P. Ribiere V. M. Bennet A. (2019). The influence of team psychological safety on team knowledge creation: a study with French and American engineering teams. J. Knowl. Manag. 23, 1157–1175. doi: 10.1108/JKM-07-2018-0420

[ref8] Cerasoli C. P. Ford M. T. (2014). Intrinsic motivation, performance, and the mediating role of mastery goal orientation: a test of self-determination theory. J. Psychol. 148, 267–286. doi: 10.1080/00223980.2013.783778, PMID: 24839727

[ref9] Cohen J. (1988). Statistical power analysis for the behavioral sciences (2nd ed.). London: Routledge.

[ref10] Crescenzi R. Gagliardi L. (2018). The innovative performance of firms in heterogeneous environments: the interplay between external knowledge and internal absorptive capacities. Res. Policy 47, 782–795. doi: 10.1016/j.respol.2018.02.006

[ref11] Eldor L. Harpaz I. (2016). A process model of employee engagement: the learning climate and its relationship with extra-role performance behaviors. J. Organ. Behav. 37, 213–235. doi: 10.1002/job.2037

[ref12] Gable P. A. Dreisbach G. (2021). Approach motivation and positive affect. Curr. Opin. Behav. Sci. 39, 203–208. doi: 10.1016/j.cobeha.2021.03.030

[ref13] Gara Bach Ouerdian E. Mansour N. al-Zahrani A. Chaari A. (2017). Promoting knowledge sharing in Tunisian KIFs through HRM practices. The mediating role of human capital and learning climate. Int. J. Hum. Resour. Manag. 30, 2321–2359. doi: 10.1080/09585192.2017.1322117

[ref14] García-Buades M. E. Ramis-Palmer C. Manassero-Mas M. A. (2015). Climate for innovation, performance, and job satisfaction of local police in Spain. Policing: An Int. J. Police Strategies Manag. 38, 722–737. doi: 10.1108/PIJPSM-02-2015-0019

[ref15] Gatsonis C. Sampson A. R. (1989). Multiple correlation: exact power and sample size calculations. Psychol. Bull. 106, 516–524. doi: 10.1037/0033-2909.106.3.516, PMID: 2813654

[ref16] Goldfarb A. Tucker C. (2019). Digital Economics. J. Econ. Lit. 57, 3–43. doi: 10.1257/jel.20171452

[ref17] Gong Y. Yao Y. Zan A. (2022). The too-much-of-a-good-thing effect of digitalization capability on radical innovation: the role of knowledge accumulation and knowledge integration capability. J. Knowl. Manag. doi: 10.1108/jkm-05-2022-0352 [Epub ahead of print].

[ref18] Haar J. O'Kane C. Cunningham J. A. (2022). Firm-level antecedents and consequences of knowledge hiding climate. J. Bus. Res. 141, 410–421. doi: 10.1016/j.jbusres.2021.11.034

[ref19] Harvey J.-F. Johnson K. J. Roloff K. S. Edmondson A. C. (2019). From orientation to behavior: the interplay between learning orientation, open-mindedness, and psychological safety in team learning. Hum. Relat. 72, 1726–1751. doi: 10.1177/0018726718817812

[ref20] Hirak R. Peng A. C. Carmeli A. Schaubroeck J. M. (2012). Linking leader inclusiveness to work unit performance: the importance of psychological safety and learning from failures. Leadersh. Q. 23, 107–117. doi: 10.1016/j.leaqua.2011.11.009

[ref21] Hwangbo Y. Shin W.-J. Kim Y. (2022). Moderating effects of leadership and innovation activities on the technological innovation, market orientation and corporate performance model. Sustainability 14:6470. doi: 10.3390/su14116470

[ref22] Inkpen A. C. (1996). Creating knowledge through collaboration. Calif. Manag. Rev. 39, 123–140. doi: 10.2307/41165879

[ref23] Janssen O. (2000). Job demands, perceptions of effort-reward fairness and innovative work behaviour. J. Occup. Organ. Psychol. 73, 287–302. doi: 10.1348/096317900167038

[ref24] Kogut B. Zander U. (1992). Knowledge of the firm, combinative capabilities, and the replication of technology. Organ. Sci. 3, 383–397. doi: 10.1287/orsc.3.3.383

[ref25] Kyndt E. Govaerts N. Claes T. De La Marche J. Dochy F. (2013). What motivates low-qualified employees to participate in training and development? A mixed-method study on their learning intentions. Stud. Contin. Educ. 35, 315–336. doi: 10.1080/0158037X.2013.764282

[ref26] Lau C. M. Ngo H. Y. (2004). The HR system, organizational culture, and product innovation. Int. Bus. Rev. 13, 685–703. doi: 10.1016/j.ibusrev.2004.08.001

[ref27] Li M. Liu H. Zhou J. (2018). G-SECI model-based knowledge creation for CoPS innovation: the role of grey knowledge. J. Knowl. Manag. 22, 887–911. doi: 10.1108/JKM-10-2016-0458

[ref28] Liu A. M. M. Chan I. Y. S. (2017). Critical role of the learning transfer climate in fostering innovation in construction. J. Manag. Eng. 33:04016050. doi: 10.1061/(ASCE)ME.1943-5479.0000482

[ref29] Liu Z. Du R. (2018). Research on relationship among entrepreneurial team knowledge heterogeneity, knowledge integration capacity and team creativity. Science Technol. Manag. Res. 8, 159–167. doi: 10.3969/j.issn.1000-7695.2018.08.023

[ref30] Loiacono E. Watson R. Goodhue D. L. (2002). WEBQUAL: a measure of website quality, 2002 marketing educators. Marketing Theory and Applications 13, 432–437.

[ref31] Lyytinen K. Yoo Y. Boland R. J. Jr. (2015). Digital product innovation within four classes of innovation networks. Inf. Syst. J. 26, 47–75. doi: 10.1111/isj.12093

[ref32] Martini A. Neirotti P. Appio F. P. (2017). Knowledge searching, integrating and performing: always a tuned trio for innovation? Long Range Plan. 50, 200–220. doi: 10.1016/j.lrp.2015.12.020

[ref33] Mehrabani S. Shajari M. (2012). Knowledge management and innovation capacity. J. Manag. Res. 4:1390. doi: 10.5296/jmr.v4i2.1390

[ref34] Men C. Luo J. Fong P. S. W. Zhong J. Huo W. (2018). Translating external knowledge to team creativity in turbulent environments: the role of absorptive capacity and knowledge integration. J. Creat. Behav. 54, 363–377. doi: 10.1002/jocb.371

[ref35] Montani F. Staglianò R. Sommovigo V. Setti I. Giorgi G. (2023). Managers' compassionate goals, innovation, and firm performance: an examination of mediating processes, and boundary conditions in small- and medium-sized enterprises. R&D Manag. 53, 97–114. doi: 10.1111/radm.12549

[ref36] Muller E. Peres R. (2019). The effect of social networks structure on innovation performance: a review and directions for research. Int. J. Res. Mark. 36, 3–19. doi: 10.1016/j.ijresmar.2018.05.003

[ref37] Nikolova I. Ruysseveldt J. V. Dam K. V. Witte H. D. (2016). Learning climate and workplace learning: Does work restructuring make a difference? J. Pers. Psychol. 15, 66–75. doi: 10.1027/1866-5888/a000151

[ref38] Nunnally J. C. Bernstein I. H. (1994). Psychometric theory 3E. McGraw-Hill.

[ref39] Peng J.-C. Chen S.-W. (2022). Learning climate and innovative creative performance: exploring the multi-level mediating mechanism of team psychological capital and work engagement. Curr. Psychol. 1–19.doi: 10.1007/s12144-021-02617-3

[ref40] Pfeffer J. Salansik G. R. (1978). A social information processing approach to job attitudes and task design. Adm. Sci. Q. 23, 224–253. doi: 10.2307/2392563, PMID: 10307892

[ref41] Pigola A. Da Costa P. R. (2022). In search of understanding about knowledge and learning on innovation performance. Scientometrics 127, 3995–4022. doi: 10.1007/s11192-022-04417-3

[ref42] Ramesh B. Tiwana A. (2001). Integrating knowledge on the web. IEEE Internet Comput. 5, 32–39. doi: 10.1109/4236.935173

[ref43] Ritala P. Huizingh E. Almpanopoulou A. Wijbenga P. (2017). Tensions in R&D networks: implications for knowledge search and integration. Technol. Forecast. Soc. Chang. 120, 311–322. doi: 10.1016/j.techfore.2016.12.020

[ref44] Schein E. H. (2004). Learning when and how to lie: a neglected aspect of organizational and occupational socialization. Hum. Relat. 57, 259–273. doi: 10.1177/0018726704043270

[ref45] Schneider B. (1990). Organizational Climate and Culture. ed. S. Zedeck Oxford: Jossey-Bass.

[ref46] Schneider B. Ehrhart M. G. Macey W. H. (2011). “Perspectives on organizational climate and culture” in APA handbook of industrial and organizational psychology, Building and developing the organization, vol. 1 (Washington, DC, US: American Psychological Association), 373–414.

[ref47] Spara E. G. (2007). Individual and unit level goal orientation as predictors of employee development. Dissertation, America(MD): University of Maryland.

[ref48] Tripathi A. Kalia P. (2022). Examining the effects of supportive work environment and organisational learning culture on organisational performance in information technology companies: the mediating role of learning agility and organisational innovation. Innovations 1-21, 1–21. doi: 10.1080/14479338.2022.2116640

[ref49] Tsui A. S. Ashford S. J. Clair L. S. Xin K. (1995). Dealing with discrepant expectations: response strategies and managerial effectiveness. Acad. Manag. J. 38, 1515–1543. doi: 10.5465/256842

[ref50] Ustalar S. A. Şanlisoy S. (2020). The impact of foreign direct investment on innovation performance: evidence from a nonlinear ARDL approach. İzmir İktisat Dergisi. 35, 77–89. doi: 10.24988/ije.202035106

[ref51] Vogt W. P. Johnson B. (2011). Dictionary of statistics & methodology: A nontechnical guide for the social sciences. Sage.

[ref52] Waheed A. Miao X. M. Waheed S. Ahmad N. Majeed A. (2019). How new HRM practices, organizational innovation, and innovative climate affect the innovation performance in the IT Industry: a moderated-mediation analysis. Sustainability 11:621. doi: 10.3390/su11030621

[ref53] Wang M.-C. Chen P.-C. Fang S.-C. (2018). A critical view of knowledge networks and innovation performance: the mediation role of firms' knowledge integration capability. J. Bus. Res. 88, 222–233. doi: 10.1016/j.jbusres.2018.03.034

[ref54] Westerberg K. Hauer E. (2009). Learning climate and work group skills in care work. J. Work. Learn. 21, 581–594. doi: 10.1108/13665620910996151

[ref55] Wu T. J. Yang Y. Yang Y. J. (2019). Does supportive organisational climate enhance employee creativity? A case of ecotec industry. J. Environ. Prot. Ecol. 20, S486–S493.

[ref56] Zhao J. (2022). Coupling open innovation: network position, knowledge integration ability, and innovation performance. J. Knowl. Econ. 1–21. doi: 10.1007/s13132-022-00932-z

[ref57] Zhong-Lin W. Kit-Tai H. Marsh H. W. (2004). Structural equation model testing: cutoff criteria for goodness of fit indices and chi-square test. Acta Psychol. Sin. 36, 186–194.

[ref58] Zobel A.-K. Lokshin B. Hagedoorn J. (2017). Formal and informal appropriation mechanisms: the role of openness and innovativeness. Technovation 59, 44–54. doi: 10.1016/j.technovation.2016.10.001

